# Domestic

**Published:** 1841-10-09

**Authors:** 


### DOMESTIC.

### HEALTH OF THE CITY

INTERMENTS *in the City and Liberties of Philadelphia, from the 25th of September, to the 2d of October.*

| Diseases. | Adults. | Children. |
|---|---|---|
| Asthma, | 1 | 0 |
| Abscess of Lungs | 0 | 1 |
| Bowel Complaint, | 0 | 3 |
| Croup, | 0 | 2 |
| Congestion of Brain, | 1 | 0 |
| Consumption of the lungs, | 8 | 2 |
| Convulsions, | 0 | 4 |
| Diarrhœa, | 1 | 2 |
| Dropsy, | 3 | 1 |
| —— Ovarian, | 0 | 1 |
| —— Head, | 0 | 2 |
| —— Breast, | 1 | 0 |
| Disease of the uterus | 1 | 0 |
| Drowned, | 1 | 1 |
| Dysentery, | 3 | 1 |
| Debility | 2 | 7 |
| Fever, | 1 | 1 |
| —— Continued, | 1 | 0 |
| —— Remittent, | 2 | 1 |
| —— Bilious, | 1 | 1 |
| —— Typhus, | 2 | 1 |
| —— Typhoid, | 0 | 2 |
| —— Scarlet, | 0 | 3 |
| Hæmorrhage from Intestines, | 1 | 0 |
| Inflammation of the Brain, | 1 | 1 |
| —— Lungs, | 0 | 1 |
| —— Stomach and Bowels, | 0 | 1 |
| —— Bowels, | 1 | 2 |
| Intemperance, | 1 | 0 |
| *Carried forward,* | 33 | 41 |

| Diseases. | Adults. | Children. |
|---|---|---|
| *Brought forward,* | 33 | 41 |
| Inanition, | 0 | 1 |
| Marasmus, | 0 | 1 |
| Malformation, | 0 | 1 |
| Mania a Potu, | 2 | 0 |
| Old age, | 1 | 0 |
| Scirrhus of liver, | 1 | 0 |
| Scrofula, | 1 | 0 |
| Small pox, | 4 | 3 |
| Still-born, | 0 | 5 |
| Suicide, | 1 | 0 |
| Spitting of blood, | 1 | 0 |
| Tabes Mesenterica, | 0 | 1 |
| Ulceration, | 1 | 0 |
| Unknown, | 0 | 1 |
| Total, 99— | 45 | 54 |

Of the above, there

| | |
|---|---|
| were under 1 year, | 21 |
| From 1 to 2 | 9 |
| 2 to 5 | 13 |
| 5 to 10 | 5 |
| 10 to 15 | 2 |
| 15 to 20 | 4 |
| 20 to 30 | 11 |
| 30 to 40 | 7 |
| 40 to 50 | 7 |
| 50 to 60 | 4 |
| 60 to 70 | 5 |
| 70 to 80 | 9 |
| 80 to 90 | 2 |
| Total, | 99 |

Of the above there were 9 from the almshouse, 10 people of colour, and 1 from the country, which are included in the total amount.

